# Concepts and Controversies in Evaluating Vitamin K Status in Population-Based Studies

**DOI:** 10.3390/nu8010008

**Published:** 2016-01-02

**Authors:** M. Kyla Shea, Sarah L. Booth

**Affiliations:** USDA Human Nutrition Research Center on Aging, Tufts University, Boston, MA 02111, USA

**Keywords:** vitamin K, epidemiology, vitamin K intake, biomarkers, review

## Abstract

A better understanding of vitamin K’s role in health and disease requires the assessment of vitamin K nutritional status in population and clinical studies. This is primarily accomplished using dietary questionnaires and/or biomarkers. Because food composition databases in the US are most complete for phylloquinone (vitamin K1, the primary form in Western diets), emphasis has been on phylloquinone intakes and associations with chronic diseases. There is growing interest in menaquinone (vitamin K2) intakes for which the food composition databases need to be expanded. Phylloquinone is commonly measured in circulation, has robust quality control schemes and changes in response to phylloquinone intake. Conversely, menaquinones are generally not detected in circulation unless large quantities are consumed. The undercarboxylated fractions of three vitamin K-dependent proteins are measurable in circulation, change in response to vitamin K supplementation and are modestly correlated. Since different vitamin K dependent proteins are implicated in different diseases the appropriate vitamin K-dependent protein biomarker depends on the outcome under study. In contrast to other nutrients, there is no single biomarker that is considered a gold-standard measure of vitamin K status. Most studies have limited volume of specimens. Strategic decisions, guided by the research question, need to be made when deciding on choice of biomarkers.

## 1. Introduction

Vitamin K is a class of structurally-similar compounds, all of which function as an enzymatic co-factor in the γ-carboxylation of vitamin K-dependent proteins [1]. While the best known vitamin K-dependent proteins are clotting proteins, vitamin K-dependent proteins are also present in many extra-hepatic tissues that have been implicated in many chronic diseases [2]. As new roles for vitamin K in health and disease emerge, so has interest in measuring vitamin K status in population-based studies. The purpose of this review is to evaluate the methods currently available to assess vitamin K status in human studies.

## 2. Vitamin K Intakes

**Figure 1:**
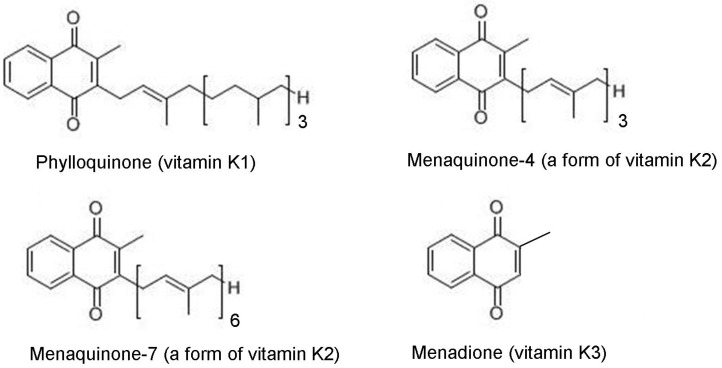
Forms of vitamin K.

A seemingly straightforward approach to estimate nutrient status is to estimate how much of the nutrient is being consumed [3]. Dietary forms of vitamin K fall into two general categories: Phylloquinone (vitamin K1) and menaquinones (collectively referred to as vitamin K2), which are comprised of at least 10 compounds (menaquinone-4 to menaquinone-13) that differ from phylloquinone in the length and saturation of their side-chain (Figure 1).

Phylloquinone is plant-based, and concentrated in green leafy vegetables and certain plant oils (1). Longer chain menaquinones (menaquinone-7–menaquinone-13) have a bacterial origin, and are primarily concentrated in animal meats and fermented foods. Menaquinone-4, which is the most similar structurally to phylloquinone, is unique among the menaquinones in that it is not produced by bacteria, but instead is either formed from phylloquinone or a pro-vitamin menadione form used in animal feed. In the human diet, menaquinone-4 is concentrated in animal meats and dairy products.

The United States Institute of Medicine’s Adequate Intake of vitamin K is set at 90 and 120 μg/day for adult women and men respectively [4]. These are based on median intakes reported in NHANES III [4]. Globally, dietary recommendations for vitamin K vary from 50 to 120 μg/day [5]. These recommendations do not differentiate phylloquinone intake from menaquinone intake. However, at the time the recommendations were set, the food composition databases from which they are based only contained the phylloquinone content of foods. Hence, the current vitamin K recommendations are based on phylloquinone, which is the primary form in Western diets [6,7,8,9,10]. As reviewed elsewhere, there is insufficient scientific knowledge at this time to determine an independent dietary recommendation for menaquinones [11].

Assessment of dietary intakes of vitamin K in population studies has relied on the use of the food frequency questionnaire (FFQ). There are many types of dietary questionnaires, which have been reviewed extensively [12,13]. In epidemiological studies, the FFQ is most commonly used because it is efficient in terms of cost and time, and imposes minimal burden on the study participant [3]. The FFQ appears to be suitable to rank individuals in terms of micronutrient intakes, but its ability to estimate absolute intakes of single nutrients is limited. The FFQ (similar to most diet questionnaires) is subjective and relies on individuals’ recall ability and perceptions, which can bias the estimates of nutrient intake [14]. Nonetheless, the FFQ has been used to estimate phylloquinone and/or menaquinone intakes as measures of vitamin K status in several studies [9,14,15,16,17,18].

The findings of population-based studies that have related phylloquinone intake to chronic disease are inconsistent (Table 1).

**Table 1:** Population-based studies of vitamin K intake and disease.

Population	Region/Cohort	Vitamin K Form and Reported Intakes	Outcome	Results	References
1836 men and 2971 women, >55 years	Rotterdam, The Netherlands (Rotterdam Study)	PK: 257 ± 116 μg/day (men); 244 μg/day (women); MK (total): 31 ± 19 μg/day (men); 33 ± 16 μg/day (women)	CHD	Highest MK tertile had lower CHD risk; PK intake not associated with CHD	[9]
807 army personnel, 39–45 years, 82% male	United States	PK: 115 ± 79 μg/day	CAC	No association	[19]
564 post-menopausal women	Utrecht, The Netherlands (PROSPECT-EPIC)	PK: 217 ± 92 μg/day; MK: 32 ± 12 μg/day	CAC	Highest MK quartile (34 ± 3 μg/day) had lower prevalence CAC; PK intake not associated with CAC prevalence	[15]
16,057 post-menopausal women	Utrecht, The Netherlands (PROSPECT-EPIC)	PK: 212 ± 100 μg/day; MK: 29 ± 13 μg/day	CHD	Higher MK intake associated with lower CHD risk; PK intake not associated with CHD	[17]
72,874 women, 38–65 years	United States (Nurse’s Health Study)	PK: 184 ± 106 μg/day	CHD	PK intake not associated with CHD once adjusted for healthy lifestyle characteristics	[20]
40,087 men, 40–75 years	United States (Physicians Health Study)	PK: 165 (67–383) μg/day (median, 5%–95%ile)	CHD	PK intake not associated with CHD once adjusted for healthy lifestyle characteristics	[8]
1112 men and 1479 women, 58 ± 9 years	Framingham, MA, United States (Framingham Offspring)	PK: 153 ± 115 μg/day (men); 171 ± 103 μg/day (women)	BMD	Higher PK intake associated with higher BMD in women, but not in men	[21]
898 women, 45–54 years	Scotland	PK: 109 ± 54 μg/day	BMD	Higher PK intake associated with higher BMD and less bone resorption	[22]
335 men and 553 women, 75 ± 5 years	Framingham, MA, United States (Framingham Heart Study)	PK: 143 ± 97 μg/day (men); 163 ± 115 μg/day (women)	BMD and hip fracture	Higher PK intake associated with lower fracture risk; not associated with BMD	[16]
72,327 women aged 38–63 years	United States (Nurse’s Health Study)	PK: 169 (41–604) μg/day (median, 1%–99%ile)	Hip fracture	Higher quintiles PK intake (≥109 μg/day) associated with lower hip fracture risk (RR: 0.70; 95% CI: 0.53, 0.93)	[23]
1605 men, 1339 women	Hong Kong	PK: 254 (157–362) μg/day (median (range), men); 239 (162–408) μg/day (median (range), women)	Hip and non-vertebral fracture	PK intake not associated with any fracture outcome	[24]
1800 women, peri-menopausal, 43–58 years	Denmark (Danish Osteoporosis Prevention Study)	PK: baseline: 67 (45–105) μg/day (median, 25%–75%iles); 5 year followup: 60 (37–99) μg/day (median, 25%–75%iles	BMD and fracture	PK intake not associated with BMD or fracture	[25]
1238 men, 1569 women, 71–75 years	Norway (Hordaland)	PK: 69 (67) μg/day (median (IQR), women); 75(62) μg/day (men); MK: 10 (7) μg/day (women); 12 (8) μg/day (men)	Hip fracture	Higher PK intake associated with lower fracture risk; no association between MK intake and fracture	[26]
625 men and women, 40–80 years	The Netherlands (PROSPECT-EPIC)	PK: 210 ± 127 μg/day; MK: 31 ± 13 μg/day	Metabolic Syndrome	Higher MK intake associated with lower prevalence MetSyn; PK intake not associated with MetSyn	[27]
510 men and women, diabetic and/or at risk for CHD, 67 ± 6 years	Spain (PREDIMED)	PK: 398 ± 201 μg/day	Insulin resistance and inflammation	Higher PK intake associated with improvements in IR and inflammation	[28]
662 men and women, 62 ± 10 years	United States (MESA)	PK: 93 ± 107 μg/day	Inflammation	No association between PK intake and inflammation	[29]
1247 men and 1472 women, 26–81 years	Framingham, MA, United States (Framingham Offspring)	PK: 139 (10 to 1975) μg/day (median (range))	Insulin resistance, sensitivity, glycemic status	Higher PK intake associated with better insulin sensitivity and glucose tolerance	[30]
11,319 men 40–64 years	Europe (EPIC-Heidelberg)	PK: 94 (71–124) μg/day (median (25%–75%ile); MK4-14: 35 (25–76) μg/day (median (25%–75%ile)	Prostate cancer	MK intake inversely associated with prostate cancer (*p*-trend = 0.06) and advanced prostate cancer (*p*-trend = 0.02)	[18]
24,340 men and women, 40–64 years	Europe (EPIC-Heidelberg)	PK 35 μg/day (median, men); MK 35 μg/day (median, men); PK 32 μg/day (median, women); MK 32 μg/day (median, women)	Cancer—lung, colorectal, breast, prostate	MK intake inversely associated with cancer incidence in men and mortality in men and women	[31]
7216 men and women, diabetic and/or at risk for CHD, 67 ± 6 years	Spain (PREDIMED)	PK: mean 356 μg/day; MK: mean 36 μg/day	Cardiovascular, cancer, all-cause mortality	Higher PK intake associated with lower cancer and all-cause mortality; MK intake not associated with mortality	[32]

**Figure 2:**
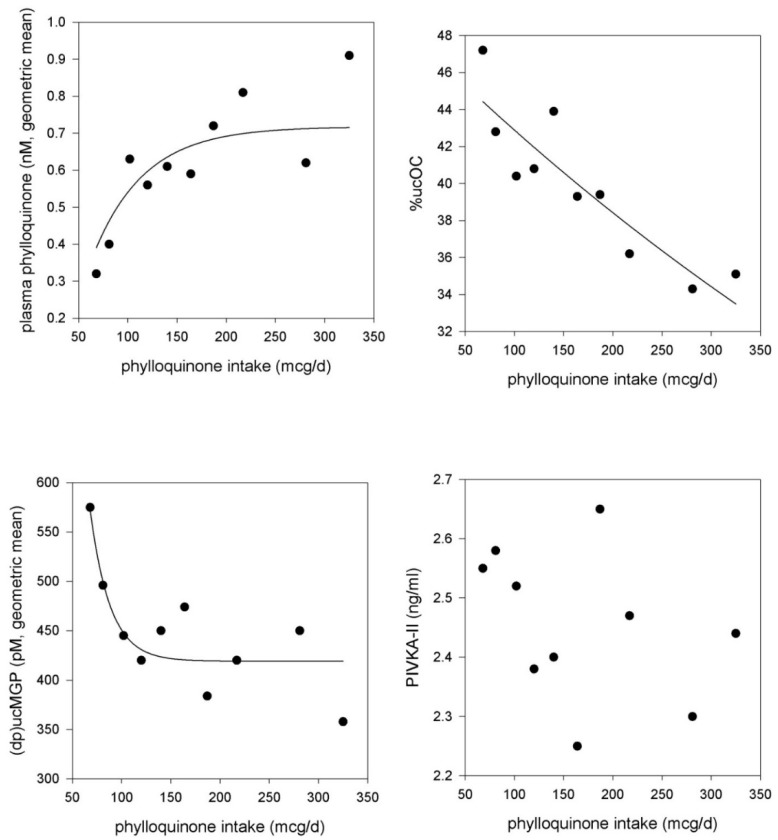
Association between food frequency questionnaire (FFQ)-estimated phylloquinone intake and circulating biomarkers of vitamin K status (at baseline) in community-dwelling older men and women participating in a phylloquinone supplementation trial [39,40]. The geometric mean of each biomarker is plotted at the median intake within each decile category. Adjustment was made for age, sex, BMI, energy intake (kcals/day), season, and triglycerides (for plasma phylloquinone).

Some found higher phylloquinone intake to be associated with higher BMD and lower fracture risk [16,26], lower cardiovascular disease (CVD) risk [8,20], improved insulin sensitivity [28,30], and lower mortality risk [32,33]. Others found phylloquinone intake was not associated with BMD [21,25], fracture [24], metabolic syndrome [27], or CVD [15,17,19,34]. The use of phylloquinone intake as the sole indicator of vitamin K status needs to be interpreted cautiously because phylloquinone intake also reflects healthy diets and lifestyles, given its concentration in green leafy vegetables and plant oils [8,20,35]. This residual confounding may not be completely eliminated when adjustments for healthy diet and/or lifestyle characteristics are made. When phylloquinone intake was estimated using the FFQ and a 5-day diet record, FFQ estimates were consistently higher, which may be due to over-reporting of vegetables [36,37]. In addition, the ability of the FFQ to accurately capture phylloquinone intakes greater than 200 μg/day is of concern. In the Framingham Offspring Study, which used the Harvard FFQ to estimate phylloquinone intake, plasma phylloquinone positively correlated with intakes up to 200 μg/d, above which the association between plasma phylloquinone and phylloquinone intake plateaued [38]. A similar pattern was observed in a separate cohort of older community-dwelling men and women in the U.S. participating in a phylloquinone supplementation trial when analyzed at baseline [39] (Figure 2).

While this plateau could reflect a saturation at the intakes >200 μg/day, circulating phylloquinone concentrations have been shown to increase to >2.0 nM in response to 500 μg/day and to >20 nM in response to 5000 μg/day of phylloquinone supplementation [39,41]. Therefore, it appears the FFQ may not be an appropriate method to estimate intakes in populations where phylloquinone intakes typically exceed 200 μg/day and results of studies doing so need to be interpreted accordingly.

Higher intakes of menaquinones were reported to be associated with less subclinical and clinical CVD, metabolic syndrome and some forms of cancer [9,15,17,18,27,31]. These associations may not be prone to confounding by a healthy diet because menaquinones are not found in foods that are typically characteristic of a healthy diet. However, the difference in total menaquinone intake between the highest and lowest categories of intake has been reported to be as narrow as ~20 μg/day [9,15,17,27]. The potential protective influence of a difference of 20 μg/day of total menaquinone is uncertain, and merits further research. It is also important to consider the food sources of menaquinones. Some cheeses rich in long-chain menaquinones are consumed in European countries more than in the US [42,43], and cheese intake itself has been associated with lower risk for type 2 diabetes, CVD, and mortality [44,45,46,47]. It is plausible menaquinone intake tracks intake of other nutrients and/or fatty acids found in cheese that have also been associated with cardiovascular disease [44,48].

The contribution of menaquinones, both in terms of forms and amounts to overall vitamin K intake varies regionally. In Western diets the relative contribution of menaquinones is thought to be much less than that of phylloquinone. However, food composition databases used in most countries do not quantify menaquinones specifically [11,49]. The most recent release of the USDA National Nutrient Database contains menaquinone-4 contents of a limited number of dairy and meat products [49]. Databases used in the Netherlands quantify individual menaquinones (menaquinone 4–10) in a limited number of foods, but are being updated [11,50]. Reported intakes of long-chain menaquinones are high in eastern Japan because *natto*, a bacterially-fermented soy food rich in menaquinone-7, is commonly eaten in that region [51]. Some have also suggested consumption of bacterially-fermented cheese, which is rich in long-chain menaquinones, is higher in the Netherlands, which may account for the long-chain menaquinone content of their diets [11]. However, a comparison of dairy intakes across EPIC study locations does not confirm this assertion [52].

Some have proposed dietary menaquinones to be more beneficial to cardiovascular and metabolic health than dietary phylloquinone [9,15,17,27]. However, the relative validity of the FFQ used in these studies was better for menaquinone than for phylloquinone [9,15,17,27]. Validity was assessed in reference to twelve 24-h recalls, but menaquinone intake validity has not yet been assessed in reference to any biomarkers. In contrast, the validity of the FFQ used to assess phylloquinone intake in these studies was poor, as acknowledged by the investigators, and the reported phylloquinone intakes were double what the current US recommendations are (means ranged from 212 to 257 μg/day) [9,17,27]. This may have blunted any ability to detect associations with dietary phylloquinone given the observed plateau effect. Furthermore, adjustment for intakes of cheese and/or other nutritional components of cheese associated with reduced CVD risk could be important but has not yet been done. Given the limitations inherent to using self-reported methods to estimate dietary intakes [3,53,54], population-based studies relating vitamin K status to chronic disease could be strengthened by the addition of vitamin K status biomarkers to dietary intake estimates.

## 3. Vitamin K Status Biomarkers

Nutritional biomarkers are biochemical measures measured in blood, urine, feces, adipose tissue or other tissues that reflect nutrient status [55]. Nutritional biomarkers are not limited by incomplete food composition databases, and are independent of recall, interviewer and social acceptance biases [55]. In contrast to dietary questionnaires which only estimate intake and generally do not account for relative bioavailability, biomarkers reflect intake, absorption and metabolism [55]. However, a thorough understanding of the biomarker’s underlying biology is required in order to consider all the physiological factors that may influence its relationship to the intake of a specific nutrient. For a comprehensive review of vitamin K bioavailability, refer to Shearer *et al*. [5]. Biomarkers can be affected by health status, including the disease outcome of interest, which can lead to misclassification and erroneous conclusions in studies that aim to identify nutritional risk factors for disease. Temporal variability needs to be considered, since many biomarkers can vary post-prandially and/or diurnally [3]. Biomarkers represent nutrient status at one point in time, which may limit extrapolation to long-term status unless repeated measures are made. In addition, biomarker analyses require rigorous laboratory standardization and quality control procedures to reduce measurement error and misclassification [56]. Nonetheless biomarkers can provide valuable estimates of nutrient status in population studies when the studies’ results are interpreted in the context of the strengths and limitations of the biomarker measured.

### 3.1. Circulating Vitamin K

Phylloquinone is the primary circulating form of vitamin K, and has been successfully measured to rank individuals’ vitamin K status in population-based and clinic-based studies worldwide (Table 2). These studies also demonstrate the large variability in circulating vitamin K in most populations.

**Table 2:** Reported circulating concentrations of vitamin K in population- or clinic-based individuals not taking vitamin K supplements (Data are mean ± SD, unless otherwise indicated.).

Participants	Region	Phylloquinone	Menaquinone	Fasted	References
Post-menopausal women: generally healthy, 52–93 years (*n* = 23)	Japan	0.22 ± 0.32 nM ^d^	MK4: 0.02 ± 0.001 nM ^d^; MK7: 0.54 ± 1.00 nM ^d^	not specified	[57]
with hip or vertebral fracture history, 66–93 years (*n* = 51)	Japan	0.21 ± 0.18 nM ^d^	MK4: non-detectable ^d^; MK7: 0.66 ± 1.00 nM ^d^	not specified	
Pre-menopausal women generally healthy, 30–49 years (*n* = 52)	Nagano, Japan	0.68 ± 0.45 nM ^d^	MK4: 0.03 ± 0.06 nM ^d^; MK7: 2.23 ± 3.12 nM ^d^	yes	[58]
Post-menopausal women generally healthy, 50–80 years (*n* = 344)		0.70 ± 0.53 nM ^d^	MK4: 0.05 ± 0.08 nM ^d^; MK7: 3.04 ± 4.32 nM ^d^	yes	
Post-menopausal women: normal BMD, 54 ± 0.8 years (*n* = 52)	Osaka, Japan	0.29 ± 0.03 nM ^d^	MK7: 2.44 ± 0.15 nM ^d^	yes	[59]
low BMD, 55 ± 1.3 years, (*n* = 19)		0.18 ± 0.02 nM ^d^	MK7 1.67 ± 0.07 nM ^d^	yes	
Post-menopausal women	Tokyo, Japan (*n* = 49; 50–84 years)	0.33 ± 0.21 nM ^d^	MK7: 2.37 ± 2.75 nM ^d^	yes	[51]
	Hiroshima, Japan (*n* = 25; 51–66 years)	0.33 ± 0.26 nM ^d^	MK7: 0.55 ± 0.83 nM ^d^	yes	
	London & Nottingham, United Kingdom (*n* = 31; 48–84 years)	0.23 ± 0.24 nM ^d^	MK7: 0.17 ± 0.09 nM ^d^	yes	
Older men, nursing home residents: normal BMD, 74 ± 10 years (*n* = 15)	Japan	0.85 ± 0.73 nM	MK7: 1.44 ± 0.85 nM		[60]
low BMD, 74 ± 11 years (*n* = 12)		0.60 ± 0.73 nM	MK7: 0.71 ± 0.35 nM		
Free living older adults: men, ≥65 years (*n* = 385)	Great Britain	0.34 (0.06–1.84) nM ^a^	NR	yes	[61]
women, ≥65 years (*n* = 493)		0.37(0.06–2.06) nM ^a^	NR		
Institution-living older adults; men, ≥65 years (*n* = 60)		0.26 (0.06–1.73) nM ^a^	NR		
women, ≥65 years (*n* = 165)		0.23 (0.06–0.89) nM ^a^	NR		
Free living older adults: men, 19–64 years (*n* = 530)	Great Britain	1.13 (0.20–8.80) nM ^a^	NR	yes	[62]
women, 19–64 years (*n* = 624)		0.81 (0.02–8.71) nM ^a^	NR		
Free living older adults: men, 65–75 years (*n* = 86)	Shenyang, China	1.88 ± 2.19 nM	NR	yes	[63]
women, 65–75 years (*n* = 92)		2.48 ± 2.88 nM	NR		
men, 60–83 years (*n* = 67)	Cambridge, United Kingdom	0.66 ± 0.75 nM	NR		
women, 60–83 years (*n* = 67)		0.73 ± 0.84 nM	NR		
Free-living women: Pre-menopausal, 31 ± 11 years (*n* = 11)	Shenyang, China	0.28 ± 0.04 nM ^b,d^	NR	yes	[64]
Post-menopausal, 68 ± 3 years (*n* = 23)		0.45 ± 0.06 nM ^b,d^	NR		
Pre-menopausal, 36 ± 11 years (*n* = 11)	Cambridge, United Kingdom	0.14 ± 0.02 nM ^b,d^	NR		
Post-menopausal, 67 ± 7 years (*n* = 31)		0.14 ± 0.01 nM ^b,d^	NR		
Pre-menopausal, 37 ± 4 years (*n* = 11)	Keneba, Gambia	0.27 ± 0.05 nM ^b,d^	NR		
Post-menopausal, 68 ± 8 years (*n* = 50)		0.16 ± 0.02 nM ^b,d^	NR		
Post-menopausal women, 57 ± 5 years (*n* = 508)	Utrecht, The Netherlands	18% non-detectable; among detectable: 1.08 ± 1.03 nM	NR	no	[65]
Hemodialysis patients, 64 ± 14 years, 63% male (*n* = 387)	Italy	0.44 ± 0.44 nM ^d^	MK4: 0.30 ± 0.33 nM ^d^; MK5: 0.45 ± 0.35 nM ^d^; MK6: 0.28 ± 0.45 nM ^d^; MK7: 0.52 ± 0.45 nM ^d^	yes	[66]
Healthy Controls, 57 ± 4 years, 70% male (*n* = 62)		0.61 ± 0.45 nM ^d^	MK4: 0.41 ± 0.38 nM ^d^; MK5: 0.58 ± 0.50 nM ^d^; MK6: 0.50 ± 0.51 nM ^d^; MK7: 0.88 ± 0.62 nM ^d^		
Patients with stage 3–5 CKD, 61 ± 14 years, 61% male (*n* = 162)	Kingston Ontario, Canada	2.1 ± 2.4 nM	NR		[67]
Patients with ESKD, 64 ± 15 years, 66% male (*n* = 44)	Kingston Ontario, Canada	1.25 ± 1.17 nM	NR		[68]
Free-living men and women: Men, 59 ± 9 years (*n* = 741)	Framingham, MA, United States	1.54 ± 2.00 nM	NR	yes	[69]
Premenopausal women, 47 ± 7 years, (*n* = 170)		1.05 ± 1.04 nM			
Postmenopausal women: Current estrogen use, 58 ± 7 years (*n* = 269)		1.46 ± 1.25 nM			
No current estrogen use, 63 ± 8 years (*n* = 424)		1.41 ± 1.54 nM			
Free-living adults: White, 62 ± 10 years, 45% male (*n* = 262)	6 communities across United States	1.3 ± 0.1 nM	NR	yes	[70]
African American, 63 ± 10 years, 47% male (*n* = 180)		1.5 ± 0.1 nM			
Hispanic, 60 ± 10 years, 51% male (*n* = 169)		1.2 ± 0.1 nM			
Chinese-American, 62 ± 10 years, 45% male (*n* = 93)		2.4 ± 0.2 nM			
Older free-living adults, 70–79 years, 38% male, 46% black (*n* = 791)	Memphis TN and Pittsburgh PA, United States	0.8 ± 0.9 nM ^c^	NR		[71]

^a^ geometric mean (inner 95% range); ^b^ geometric mean ± SEM; ^c^ median ± interquartile range; ^d^ reported as ng/mL, converted to nmol/L by multiplying ng/mL by 2.22; NR: Not reported.

However, the number of studies that have evaluated circulating phylloquinone in relation to chronic disease is relatively few compared to the studies that assessed dietary vitamin K intake. Higher circulating phylloquinone has been associated with less bone loss and fracture [66,69,72], less osteoarthritis [71,73,74], and less coronary calcium progression is some [75] but not all [65] cohorts. Circulating concentrations of phylloquinone are 50 to 25,000 times lower than other fat-soluble nutrients, which has historically presented technological challenges in its measurement [76]. Sensitive HPLC and mass spectrometry assays have been developed to measure phylloquinone in blood but there is considerable variability in the separation techniques and assay standards used, some of which leads to erroneous reporting. However external quality assurance programs are now available to standardize assays used from one laboratory to the next and monitor inter-laboratory variation, and any study measuring circulating phylloquinone should be an active member of these programs to verify accuracy of the measures reported [76].

**Figure 3:**
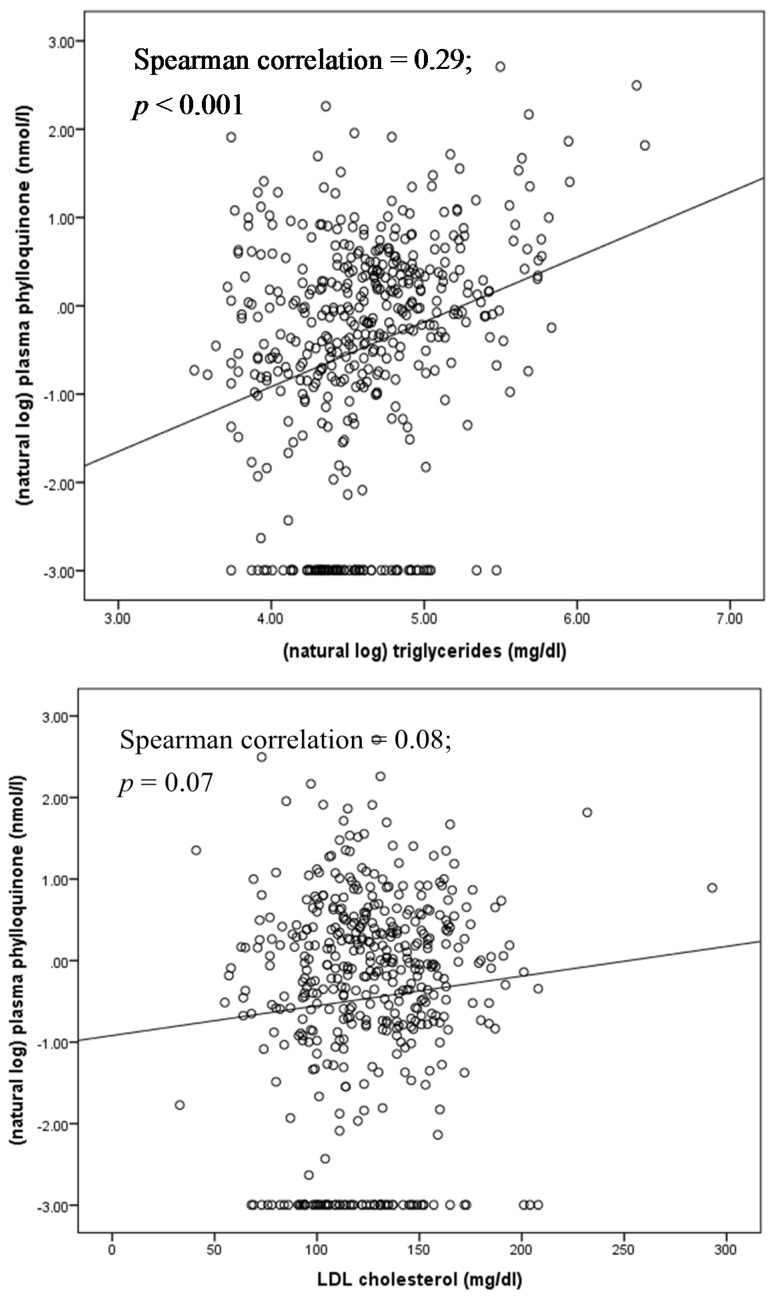

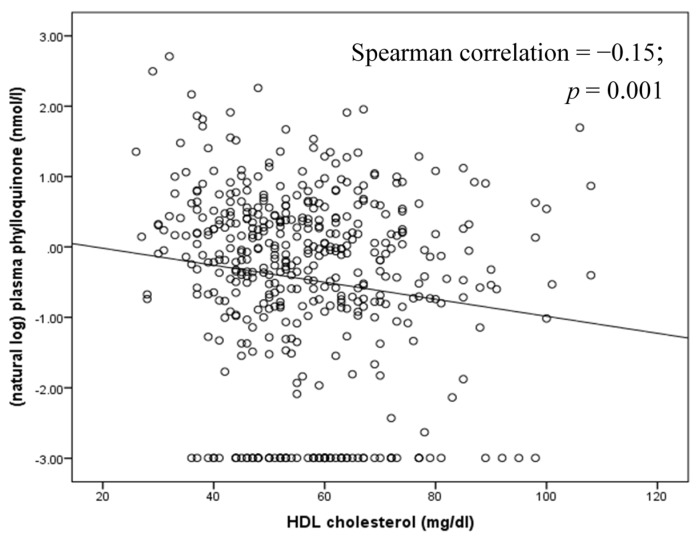
Correlation between circulating phylloquinone and lipids at baseline in community-dwelling older men and women participating in a phylloquinone supplementation trial [39,40].

Phylloquinone is transported on triglyceride-rich lipoproteins in circulation, with smaller fractions carried on HDL and LDL cholesterol [77] (Figure 3).

Circulating phylloquinone responds to changes in dietary phylloquinone intake [78,79], and concentrations peak 6–10 h post-prandially [80]. There is also some indication that the response of circulating concentrations of phylloquinone vary with the type of meal pattern [81]. Given the physiology underlying phylloquinone absorption and transport, circulating phylloquinone should be measured in fasting samples and corrected for triglycerides to better reflect overall nutritional status. In population-based studies, adjustment for triglycerides strengthened associations between circulating phylloquinone and bone mineral density [69] and coronary calcium progression [75]. In studies that have not adjusted plasma phylloquinone for triglycerides, the data are more difficult to interpret. For example, in a sub-study of the Dutch Prospect cohort, post-menopausal women with the higher plasma phylloquinone had a higher prevalence of coronary calcium (CAC, an indicator of subclinical CVD) [65]. However, the plasma samples from which phylloquinone was measured were not obtained in a fasted state and triglyceride measures were unavailable. The investigators adjusted for cholesterol instead, but cholesterol is weakly correlated with circulating phylloquinone (Figure 3), and does not reflect its absorption. Since hypertriglyceridemia is a risk factor for CVD [82], it is plausible the phylloquinone tracked elevated triglycerides.

There is currently no established threshold of plasma/serum phylloquinone that indicates insufficiency or deficiency. When the Adequate Intake is met in controlled feeding studies, circulating phylloquinone concentrations approximate 1.0 nM [83]. However, it is not currently known if the Adequate Intake is sufficient to meet all physiological needs, especially health outcomes not related to coagulation [2]. As a corollary, it is not known if 1.0 nM of circulating phylloquinone is similarly sufficient. As such, low circulating phylloquinone has not been consistently defined in the scientific literature [71,72,73,74,75], making it difficult to clarify a threshold for insufficiency. Additional research in this area is needed.

Circulating phylloquinone appears to be influenced by more than dietary vitamin K intake and triglycerides [38,70,84], and much of the variability remains to be explained. In a multi-ethnic US cohort, race-ethnicity was identified as a significant predictor of serum phylloquinone with Chinese-Americans and African-Americans having higher concentrations than Caucasians and Hispanics [70]. In a primarily Caucasian cohort, plasma phylloquinone was not found to be significantly heritable [84]. However, a subsequent GWAS analysis of Caucasian participants in three US cohorts identified multiple candidate genes as potential determinants of circulating phylloquinone [85]. Since none of the variants achieved significance at the GWA level of <1 × 10^8^ larger studies are needed to confirm these findings, and more diverse cohorts are needed to expand these findings to include other race/ethnic groups. This requires more studies that measure plasma/serum phylloquinone, which to date has not been a common biomarker in large-scale population studies.

Studies reporting circulating menaquinones as a biomarker of vitamin K status are more limited (Table 2) because menaquinones are generally not detected in circulation unless supplements are taken or large quantities of menaquinone-rich foods are consumed. In women from Nagano Japan, circulating menaquinone-7 is reported to exceed 10 nM, which likely reflects natto consumption in that region. In these same women, the average concentration of menaquinone-4 was 0.2 nM, although >50% had non-detectable concentrations despite use of highly sensitive instrumentation [58]. In two studies using less sensitive HPLC methods with post-column reduction and fluorescence detection (lower limit of detection ~0.1 ng/mL), menaquinone-4 was detected in only 10% of 105 postmenopausal women from Japan or the United Kingdom [51] and in none of 440 postmenopausal Canadian women with osteopenia, half of whom were consuming daily doses of 5 mg of phylloquinone [41] (Booth *et al.*, unpublished data). These observational data are also consistent with a recent intervention study that showed single or consecutive oral administration of menaquinone-4 failed to increase serum menaquinone-4 concentrations [86]. At odds to the collective observations of others, in a study conducted in Italy, Fusaro *et al*. reported relatively high plasma concentrations of menaquinones 4–7 in chronic kidney disease patients and healthy controls not taking supplements, with chronic kidney disease patients having lower average menaquinone and phylloquinone concentrations [66]. In this same study, low concentrations of menaquinone-4 and menaquinone-7 were associated with significantly higher odds of aortic artery and iliac artery calcification respectively, and low menaquinone-5 concentrations were found to be associated with lower odds for abdominal aorta calcification. The concentration of menaquinone-5 was reported to be relatively equal to that of circulating phylloquinone, even though menaquinone-5 is not found in many foods and is rarely synthesized by bacteria [49]. No other study has reported such high concentrations of circulating menaquinone concentrations among individuals not taking menaquinone supplements and there are no other studies that have reported detectable menaquinone-5 concentrations in circulation despite concerted efforts to find circulating menaquinones using very sensitive mass spectrometry instrumentation [87]. Given the contradictory associations of menaquinone-5 with aortic calcification does not fit our current understanding of the role of vitamin K-dependent proteins in calcification, there are sufficient doubts regarding the validity of these findings. Until the findings of the study by Fusaro *et al*. [66] are independently replicated, use of circulating menaquinones as a measure of vitamin K status in population studies is uncertain.

### 3.2. Undercarboxylated Vitamin K-Dependent Proteins

When there is insufficient vitamin K due to low vitamin K intake or vitamin K antagonism with oral anticoagulants such as warfarin, the post-translational carboxylation of vitamin K dependent proteins is reduced, which means the undercarboxylated (inactive) fractions of these proteins increases. Of the known vitamin K-dependent proteins, clotting proteins are the most recognized [1]. As reviewed elsewhere [88] prothrombin time, also expressed as international normalized ratio, is a routine clinical assay that can reflect clinical deficiency of vitamin K. However, these tests are non-specific, have low sensitivity for detecting low vitamin K status [89] and do not reflect intakes in generally healthy adults (Figure 2), so are not used in population studies as a measure of vitamin K status. Undercarboxylated prothrombin, known as PIVKA-II (proteins induced in vitamin K absence or antagonism-factor II), is measurable in circulation, and PIVKA-II concentrations change in response to dietary vitamin K depletion [83] and warfarin use [90]. Commercially-available PIVKA-II assays have low sensitivity for detecting variation in normal vitamin K intakes, which limits its utility in ranking individuals in population studies. One exception is in patients with chronic kidney disease, given the high prevalence of vitamin K deficiency in this patient population. PIVKA-II is not affected by reduced kidney function so circulating concentrations should not be influenced by the disease itself [68].

Osteocalcin (OC) is a vitamin K-dependent protein synthesized exclusively in bone during bone formation. OC is also detectable in serum and is used as a bone formation biomarker [91,92,93]. During dietary vitamin K depletion, the undercarboxylated fraction of OC (ucOC) increases whereas it decreases in response to vitamin K supplementation [83,94]. UcOC is detectable in circulation even when vitamin K intakes are sufficient to maintain coagulation, suggesting the storage of vitamin K in extra-hepatic organs is secondary to hepatic storage, as demonstrated in animal models [95,96]. For this reason, ucOC reflects vitamin K intake more so than PIVKA-II and is thought to be a more sensitive indicator of vitamin K status in community-based individuals (Figure 2). There are two immunoassay methods available to measure undercarboxylated OC: the hydroxyapatite binding assay or a commercially available immunoassay, which measures fully undercarboxylated OC directly (Takara Inc., Kyoto, Japan) [91,97]. More recently, a mass spectrometric immunoassay was developed that can provide qualitative and relative percent abundance information on the molecular variants of OC present in serum [94]. The absolute concentration of ucOC is positively correlated with total OC (*r* = 0.78) (Table 3). Because of this, when serum ucOC is used as a measure of vitamin K status it should be expressed as a% of total OC (%ucOC; when measured using a hydroxyapatite binding assay) or as a ratio to the carboxylated OC when measured directly (using immunoassay) [91], but not all studies have done so [98].

**Table 3:** Correlations among phylloquinone intake and biomarkers of vitamin K status in community-dwelling primarily Caucasian older adults (*n* = 443). Data are presented as Pearson correlation coefficients (*p*-value).

	Phylloquinone Intake (µg/Day) ^a,b^	Plasma Phylloquinone (nM) ^a,c^	PIVKA (ng/mL) ^d^	%ucOC ^e^	ucOC (ng/mL) ^e^	Total OC (ng/mL) ^e^	(dp)ucMGP (pM) ^a,f^
plasma phylloquinone (nM) ^a,c^	0.17 (<0.001) ^h,i^						
PIVKA (ng/mL) ^d^	−0.05 (0.30) ^h^	−0.17 (<0.001) ^i^					
%ucOC ^e^	−0.14 (0.003) ^h^	−0.23 (<0.001) ^i^	0.08 (0.11)				
ucOC (ng/mL) ^e^	−0.06 (0.19) ^h^	−0.18 (<0.001) ^i^	0.04 (0.42)	0.78 (<0.001)			
Total OC (ng/mL) ^e^	0.02 (0.64) ^h^	−0.08 (0.09) ^i^	−0.02 (0.74)	0.41 (<0.001)	0.84 (<0.001)		
(dp)ucMGP (pM) ^af^	−0.14 (<0.001) ^h^	−0.32 (<0.001)	−0.06 (0.24)	0.26 (<0.001)	0.22 (<0.001)	0.08 (0.08)	
Total MGP (ng/mL) ^g^	0.08 (0.10) ^h^	0.04 (0.46) ^i^	−0.06 (0.24)	−0.10 (0.03)	−0.03 (0.52)	0.05 (0.29)	0.29 (<0.001)

^a^ natural log transformed to reduce skewness; ^b^ estimated using the Harvard Food Frequency Questionaire [39]; ^c^ measured using reverse-phase HPLC [39]; ^d^ measured using enzyme-linked immunoassay (ELISA) (Diagnostica Stago) [99]; ^e^ measured using radioimmunoassay [39,91]; ^f^ measured using sandwich ELISA [100,101]; ^g^ measured using radioimmunoassay [40,102]; ^h^ *n* = 438; ^i^ adjusted for triglycerides.

Because osteocalcin is synthesized in bone, the early studies of %ucOC as a functional indicator of vitamin K status focused on bone. Observational evidence suggested lower %ucOC to be associated with higher bone mineral density and reduced hip fracture risk [69,103,104,105,106], leading to the hypothesis that reducing %ucOC with vitamin K supplementation would decrease age-related bone loss. While some randomized trials reported a beneficial effect of vitamin K on bone health [41,107,108], others did not, even though vitamin K supplementation effectively reduced %ucOC [39,109,110,111,112]. Hence, the relevance of %ucOC to bone health is equivocal, as previously reviewed [97].

There has been recent interest in ucOC and its putative role in regulating glucose homeostasis. This theory was developed based on animal models which found injection of ucOC reduced blood glucose and improved insulin sensitivity in mice [113,114]. Several [115,116,117,118,119,120], but not all [117,121], studies that sought to extrapolate this finding to humans, found circulating ucOC to be inversely correlated to measures of insulin resistance. This could suggest a protective role for ucOC and therefore infer a detrimental role for vitamin K in metabolic disease because vitamin K promotes OC carboxylation. Since the primary dietary sources of vitamin K are green leafy vegetables and vegetable oils, this would represent a paradigm shift with respect to health benefits of vitamin K-rich foods. However, these studies did not correct for total osteocalcin, hence did not differentiate between an overall bone effect, as indicated by changes in the osteocalcin molecule, and a vitamin K effect, which would have been isolated if expressed as %ucOC. The limitations of these earlier population studies have been reinforced by the results of a recent meta-analysis, which found total OC and ucOC to be similarly inversely associated with fasting plasma glucose and glycosylated hemoglobin. Collectively, these suggest that the OC, but not its carboxylation status, hence vitamin K status, may be relevant to insulin resistance [122].

Matrix gla protein (MGP) is a vitamin K-dependent protein that functions as a calcification inhibitor in vascular tissue and cartilage [123]. In addition to being post-translationally carboxylated, MGP is also phosphorylated [124]. Assays that measure different forms of MGP in plasma are available [100], and the different circulating species appear to be differentially associated with health outcomes related to calcification (Supplementary Table S1). Only the dephosphorylated undercarboxylated form ((dp)ucMGP) responds to vitamin K supplementation [100,101,125,126]. When plotted against FFQ-estimated vitamin K intake, (dp)ucMGP decreased up an intake of 100–150 μg/day at which point the association plateaued (Figure 2), which may reflect the limitations of estimating vitamin K intake using FFQs previously discussed. (Dp)ucMGP has been suggested to be a functional indicator of vitamin K status in tissues that utilize MGP [100,127], such that higher amounts of (dp)ucMGP reflect lower vitamin K status. Several [128,129,130,131,132,133,134], but not all [135,136] population-based studies found higher plasma (dp)ucMGP was associated with more arterial calcification, arterial stiffness (which is positively associated with calcification) and CVD. In a post hoc analysis of a randomized controlled trial that found phylloquinone supplementation reduced CAC progression over 3 years in older men and women, dp-ucMGP was reduced by phylloquinone supplementation, but the change in (dp)ucMGP did not correlate with change in CAC [101]. Recently menaquinone-7 was reported to reduce plasma (dp)ucMGP and have a beneficial effect on arterial stiffness in post-menopausal women. At baseline (dp)ucMGP was positively correlated with stiffness, but the investigators did not report if changes in (dp)ucMGP correlated with changes in stiffness [137]. Because the majority of studies that measured (dp)ucMGP were done in primarily Caucasian groups (Supplementary Table S1), the relevance of (dp)ucMGP to vitamin K status and health outcomes in non-Caucasian groups merits investigation.

Similar to the situation with osteocalcin, the amount of (dp)ucMGP in circulation also depends on the total amount of MGP available [91,101]. It may be inappropriate, therefore, to extrapolate the association of higher (dp)ucMGP with more adverse health outcomes as being related to vitamin K insufficiency because the associations may be related to overall MGP status. For example, total MGP increases with age, independent of vitamin K intake [102], and given that age is an independent predictor of CVD and other chronic diseases, MGP may simply be a strong biomarker for age. It is also plausible that diseases characterized by calcification and/or cardiac dysfunction affect the synthesis of MGP because calcium as well as cardiac overload can promote MGP expression [138,139,140]. This could suggest elevated MGP is a consequence of CVD rather than causal. Much remains to be understood about the physiology underlying MGPs role in health and disease, which cannot be ascertained using observational studies. It is premature to conclude increasing MGP carboxylation with vitamin K will reduce risk for CVD and other outcomes related to calcification until this is tested using well-designed clinical trials.

### 3.3. Urinary Biomarkers

In addition to the blood measures, methods have been developed to measure urinary biomarkers of vitamin K metabolism. ү-Carboxyglutamic acid (gla) is an indicator of the turnover of all vitamin K-dependent proteins. Some [83,141], but not all [79,142] studies found urinary gla decreased when vitamin K intake is decreased. As vitamin K is catabolized, 5 carbon and 7 carbon aglycone metabolites are produced, which are water soluble and excreted in urine. These metabolites increase in response to vitamin K supplementation [143] and repletion, with a concomitant decrease in response to vitamin K depletion [143]. Menadione (vitamin K3; Figure 1) is the naphthoquinone ring metabolite of vitamin K that is thought to be an intermediary in the tissue-specific conversion of phylloquinone to menaquinone-4. Menadione is detectable in urine [144] and was found to change in response to vitamin K depletion and repletion more than urinary gla [141]. Because these urinary measures ideally require 24 h urine collection, their utility in clinic-based or in large-scale population-based studies is very limited. To the best of our knowledge, there are no studies that have examined the association of any of these measures with health outcomes for which a role for vitamin K has been suggested.

### 3.4. Interrelatedness of Vitamin K Status Biomarkers

Although there are multiple biomarkers available to estimate vitamin K status, it is apparent that no single biomarker is valid for use across all population-based studies. Since most population-based studies have limited volume of specimens, strategic decisions need to be made when deciding on choice of biomarkers.

**Figure 4:**
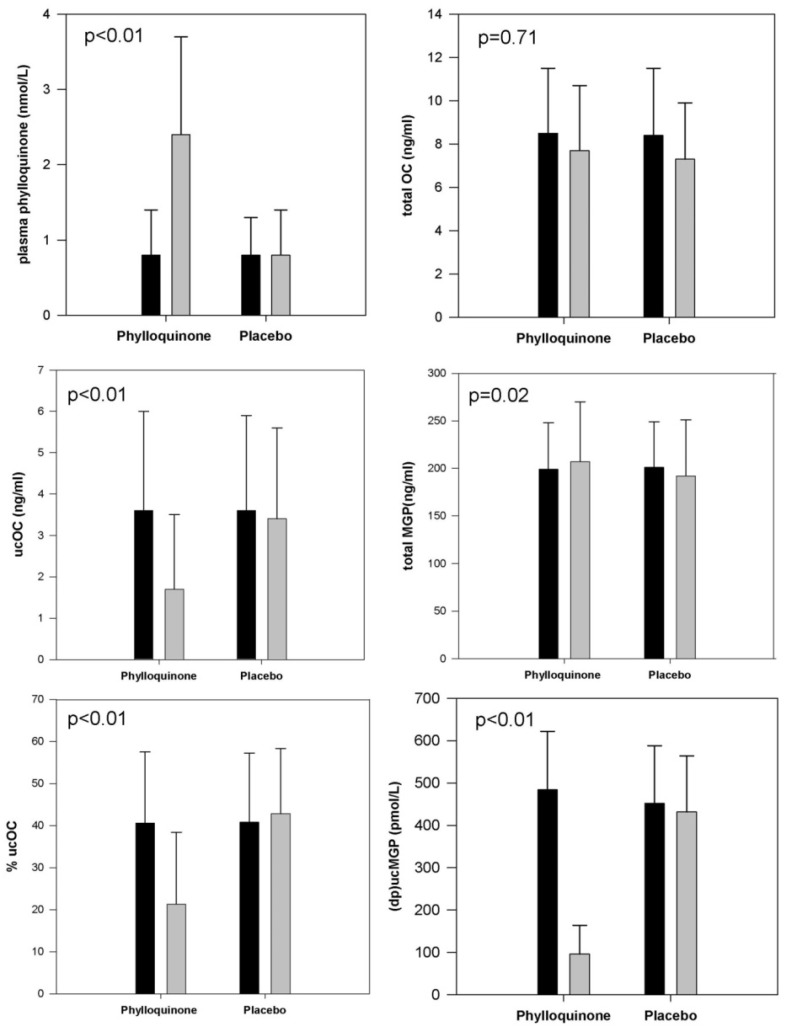
Circulating biomarkers at baseline (■) and after 3 years (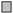) of supplementation with 500 μg/day phylloquinone (*n* = 229) or placebo (*n* = 223) in primarily Caucasian community-dwelling men and women 65–80 years old. (Because of skewed distributions, plasma phylloquinone and (dp)ucMgp are presented as median values with error bars representing inter-quartile ranges. Otherwise data are presented as means with error bars representing standard deviations. *p*-values reflect the between-group difference for change in the biomarker in response to phylloquinone supplementation *versus* placebo).

After three years of supplementation with 500 µg/day of phylloquinone, plasma phylloquinone increased by >100% and %ucOC (and ucOC ng/mL) and (dp)ucMGP decreased by 50%–80% (Figure 4) [39,40]. The total MGP decreased 3.5% in the placebo group and increased 3.5% in the phylloquinone supplemented group, and the between group difference in this change reached statistical significance. However, the biological relevance of this difference is uncertain.

**Figure 5:**
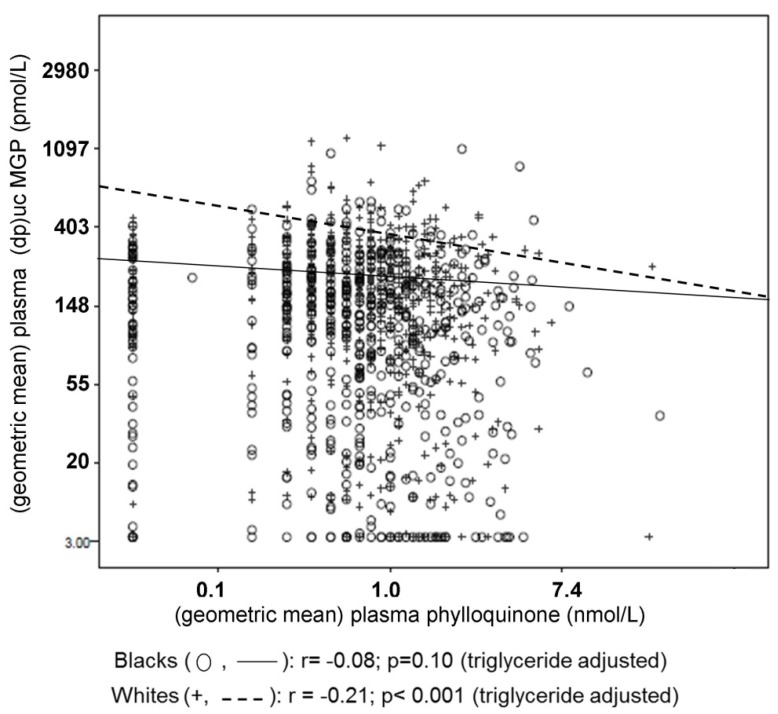
Correlation between circulating phylloquinone and (dp)ucMGP in black (*n* = 507) and white (*n* = 570) men and women 70–79 years old.

PIVKA-II amounts were overall within the normal range at baseline [99], hence were not examined in response to supplementation given the low sensitivity of PIVKA-II to vitamin K supplementation in healthy individuals. Relative changes of urinary gla were more subtle compared to changes in circulating phylloquinone or undercarboxylated vitamin K-dependent proteins [83,141]. In this same cohort [39,40] observed at baseline, with the exception of PIVKA-II, circulating biomarkers of vitamin K significantly correlated with phylloquinone intake and with one another, but the correlations were overall not strong (Table 3). Unfortunately there are no urinary measures of vitamin K status available for comparison in population studies. Interestingly, in a racially diverse cohort, (dp)ucMGP was significantly correlated with plasma phylloquinone in whites but not in blacks (Figure 5) [145].

This may suggest the different measures of vitamin K status are influenced by physiological factors in addition to vitamin K. At this time, a combination of circulating concentrations of phylloquinone (and menaquinones when detectable), preferably corrected for circulating triglycerides, with an undercarboxylated non-coagulation protein, such as %ucOC (which can be corrected for the non-dietary influences on total protein concentrations), or (dp)ucMGP, would provide the most comprehensive approach to measure vitamin K status in population-based studies at this time, and allow for suitable ranking.

## 4. Conclusions

In summary, despite the multiple measures available, population- and clinic-based studies of vitamin K status are challenged by the lack of a single gold-standard measure. Dietary intake assessment by FFQ is convenient and easily implemented. Food composition databases are being expanded to include multiple menaquinone forms, so dietary questionnaires will no longer be limited to phylloquinone intake. Because menaquinones are generally not detected in circulation unless very large amounts are consumed, correlating menaquinone intake to circulating levels is problematic. While the use of biomarkers to estimate vitamin K status is not subject to the same limitations as the use dietary intake measures [3,53,54], there are limitations to each of the available biomarkers that need to be considered when studies are designed and interpreted. Circulating phylloquinone is carried on triglyceride-rich lipoproteins, which necessitates the use of fasting samples and concomitant measurement of triglycerides to reduce confounding. The amount of undercarboxylated vitamin K-dependent proteins in circulation depends on the total amount of protein, in addition to vitamin K availability. If these measures are not corrected for the total amount of the protein under study, they may reflect total protein status as well as vitamin K status which can confound the interpretation of the results. At this time, none of the urinary measures have been applied large cohorts so their utility epidemiologically is uncertain. Given the strengths and limitations of the available measures, and their modest inter-relatedness, vitamin K status may be estimated more accurately if multiple biomarkers, or biomarkers in combination with dietary intake are used.

## Supplementary Materials

Supplementary materials can be accessed at: http://www.mdpi.com/2072-6643/7/12/5554/s1.

## Abbreviations

**BMD**: bone mineral density, **CAC**: coronary artery calcium, **CHD**: coronary heart disease, **(dp)ucMGP**: dephosphorylated undercarboxylated matrix gla protein, **ELISA**: enzyme-linked immunoassay, **EPIC**: European Prospective Investigation into Cancer and Nutrition Study, **FFQ**: food frequency questionnaire, **HPLC**: high performance liquid chromatography, **IR**: insulin resistance, **MESA**: Multi-ethnic Study of Atherosclerosis, **MGP**: matrix gla protein, **MK**: menaquinone, **PIVKA-II**: proteins induced in vitamin K absence or antagonism-factor II, **PK**: phylloquinone, **ucOC**: undercarboxylated osteocalcin, **%ucOC**: percent undercarboxylated osteocalcin.
