# Supplementary material for: Concepts and Controversies in Evaluating Vitamin K Status in Population-Based Studies
Source: Nutrients. 2016 Jan 2;8(1):8. doi: 10.3390/nu8010008 (PMC4728622; doi:10.3390/nu8010008)
Supplement: Supplementary file 1 [file nutrients-08-00008-s001.docx]

**Supplementary Materials: Concepts and Controversies in Estimating Vitamin K Status in Population-based Studies**

M. Kyla Shea and Sarah L. Booth

**Table S1.** Studies of circulating forms of MGP and health outcomes related to vascular calcification.

| **Participants** | **Design** | **Outcome(s)** |  | **MGP Form Studied and Results** | |  | **References** |
| --- | --- | --- | --- | --- | --- | --- | --- |
|  |  |  | **Total ucMGP** | **(dp)ucMGP** | **(dp)cMGP** | **Total MGP** |  |
| various clinical groups (*n* ≈ 20–25) & controls (*n* = 200) | Case-control | CVD, bone, T1DM, control | NM (not measured) | NM | NM | ↑ in advanced CVD, T1DM | [1] |
| 115 with coronary heart disease (CHD) | Cross-sectional | coronary artery calcium (CAC) | NM | NM | NM | Inversely associated with CAC | [2] |
| 120 hemodialysis (HD) patients & 41 age-matched healthy controls | Case-control & cross-sectional | arterial stiffness | ↓ in HD patients; inversely associated with one stiffness measure | NM | NM | NM | [3] |
| 165 healthy subjects, 30 angioplasty, patients, 25 aortic stenosis (AS) patients, 52 HD patients, 10 calciphylaxis patients | Case-control |  | ↓ in all patient groups, especially HD and calciphylaxis patients | NM | NM | NM | [4] |
| 40 HD patients; 48 healthy controls >60 years | Case-control , cross-sectional | HD and CAC | ↓ in HD patients, inversely associated with CAC | NM | NM | NM | [5] |
| 839 outpatients with stable CVD; stratified by T2DM | Cross-sectional | mitral valve calcification (MVC), AS | Positively associated with MVC in T2DM; inversely associated with MVC in non-T2DM; no association with AS | NM | NM | NM | [6] |
| 833 outpatients with stable CVD (same cohort as above) | Prospective | CVD and all-cause mortality | Inversely associated with CVD & all-cause mortality | NM | NM | NM | [7] |
| 191 patients with aortic valve disease ; 35 controls | Case-control |  | ↓ in aortic valve disease patients | NM | NM | NM | [8] |
| 107 patients with chronic kidney disease | Cross-sectional, prospective | aortic calcification and mortality | NM | Positively associated with kidney disease severity and all-cause mortality | NM | NM | [9] |
| 36 patients with hypertension | Cross-sectional | total arterial calcium *Z*-score | Positively associated with calcium Z-score | NM | NM | NM | [10] |
| 147 patients with  AS 93 sex-matched controls >60 years | Case-control, cross-sectional prospective | left ventricular ejection fraction (LVEF); all-cause mortality | NM | ↑ in AS patients; inversely associated with LVEF, positively associated with all-cause mortality | ↑ in AS patients; no association with LVEF or all-cause mortality | NM | [11] |
| 188 HD patients; 98 age-matched healthy controls | Case-control, cross-sectional prospective | vascular calcification, CVD mortality and all-cause mortality | NM | ↑ in HD; no association with vascular calcification, CVD or all- cause mortality in HD | ↑ in HD; associated with ↓ vascular calcification and with ↓ CVD and all-cause mortality in HD | NM | [12] |
| 179 patients with chronic heart failure; 33 sex and age-matched controls | Case- control, cross-sectional, prospective | diastolic dysfunction; mortality | NM | ↑ in HF patients; positively associated with diastolic dysfunction and mortality | ↑ in HF patients; positively associated with diastolic dysfunction, no association with mortality | NM | [11] |
| 438 community-dwelling adults | Cross-sectional, prospective | CAC | NM | No association | NM | NM | [13] |
| 200 postmenopausal healthy women in PROSPECT (Dutch population-based study) | Cross-sectional | CAC | No association | No association | Inversely associated with CAC |  | [14] |
| 72 postmenopausal women at risk for CVD | CC and cross-sectional | carotid artery stenosis, carotid intima-medial thickness (cIMT) | NM | NM | ↑ in carotid stenosis; among those with stenosis: ↑ in T2DM & hypertension; associated with ↓ cIMT | NM | [15] |
| 518 participants in EPIC-Netherlands with T2DM (Dutch population-based study) | Prospective | CVD & subtypes (CHD, HF, stroke); | ↑ associated with ↑ CVD, ↑ PAD, ↑ HF | No association with any outcome | No association with any outcome | NM | [16] |
| 2940 participants in EPIC-Netherlands (Dutch population-based study) | Prospective case cohort | CHD, stroke | NM | No association with CHD or stroke | NM | NM | [17] |
| 577 participants in LASA (Dutch population-based study) | Prospective | CVD | NM | Positively associated with CVD | No association | NM | [18] |
| 799 patients who had myocardial infarction | Prospective | CVD and total mortality | NM | Positively associated with CVD and total mortality | ↑ associated with ↑ CVD and total mortality | NM | [19] |
| 1087 participants in Czech post-MONICA (population –based study) | Cross-sectional | arterial stiffness | NM | Positively associated with arterial stiffness | NM | NM | [20] |
| 2318 FLAMENGHO participants (population-based study, Belgium) | Prospective | CVD, CVD mortality, total mortality | NM | Positively associated with CVD mortality, but inversely associated with coronary events | NM | NM | [21] |
| 1035 SKIPOGH participants (population-based study, Switzerland) | Cross-sectional | Renal resistive index | NM | Positively associated with renal resistive index | NM | NM | [22] |
| 1001 SKIPOGH participants (population-–based study, Switzerland) | Cross-sectional | arterial stiffness | NM | Positively associated with arterial stiffness | NM | NM | [23] |

Abbreviations: AS: aortic stenosis; CAC: coronary artery calcium; CHD: coronary heart disease; cIMT: carotid intima medial thickness; CVD: cardiovascular disease; HD: hemodialysis; HF: heart failure; LVEF: left ventricular ejection fraction; MVC: mitral valve calcification; NM: not measured; RA: rheumatoid arthritis; T1DM: type 1 diabetes mellitus; T2DM: type 2 diabetes mellitus.

**Reference**

1. Braam, L.A.; Dissel, P.; Gijsbers, B.L.; Spronk, H.M.; Hamulyak, K.; Soute, B.A.; Debie, W.; Vermeer, C. Assay for human matrix Gla protein in serum: Potential applications in the cardiovascular field. *Arterioscler. Thromb. Vasc. Biol.* **2000**, *20*, 1257–1261.
2. Jono, S.; Ikari, Y.; Vermeer, C.; Dissel, P.; Hasegawa, K.; Shioi, A.; Taniwaki, H.; Kizu, A.; Nishizawa, Y.; Saito, S. Matrix Gla protein is associated with coronary artery calcification as assessed by electron-beam computed tomography. *Thromb. Haemost*. **2004**, *91*, 790–794.
3. Hermans, M.M.; Vermeer, C.; Kooman, J.P.; Brandenburg, V.; Ketteler, M.; Gladziwa, U.; Rensma, P.L.; Leunissen, K.M.; Schurgers, L.J. Undercarboxylated matrix GLA protein levels are decreased in dialysis patients and related to parameters of calcium-phosphate metabolism and aortic augmentation index. *Blood Purif*. **2007**, *25*, 395–401.
4. Cranenburg, E.C.; Vermeer, C.; Koos, R.; Boumans, M.L.; Hackeng, T.M.; Bouwman, F.G.; Kwaijtaal, M.; Brandenburg, V.M.; Ketteler, M.; Schurgers, L.J. The circulating inactive form of matrix Gla Protein (ucMGP) as a biomarker for cardiovascular calcification. *J. Vasc. Res.* **2008**, *45*, 427–436.
5. Cranenburg, E.C.; Brandenburg, V.M.; Vermeer, C.; Stenger, M.; Muhlenbruch, G.; Mahnken, A.H.; Gladziwa, U.; Ketteler, M.; Schurgers, L.J. Uncarboxylated matrix Gla protein (ucMGP) is associated with coronary artery calcification in haemodialysis patients. *Thromb. Haemost*. **2009**, *101*, 359–366.
6. Parker, B.D.; Schurgers, L.J.; Vermeer, C.; Schiller, N.B.; Whooley, M.A.; Ix, J.H. The association of uncarboxylated matrix Gla protein with mitral annular calcification differs by diabetes status: The Heart and Soul study. *Atherosclerosis* **2010**, *210*, 320–325.
7. Parker, B.D.; Schurgers, L.J.; Brandenburg, V.M.; Christenson, R.H.; Vermeer, C.; Ketteler, M.; Shlipak, M.G.; Whooley, M.A.; Ix, J.H. The associations of fibroblast growth factor 23 and uncarboxylated matrix Gla protein with mortality in coronary artery disease: The Heart and Soul Study. *Ann. Intern. Med*. **2010**, *152*, 640–648.
8. Koos, R.; Krueger, T.; Westenfeld, R.; Kuhl, H.P.; Brandenburg, V.; Mahnken, A.H.; Stanzel, S.; Vermeer, C.; Cranenburg, E.C.; Floege, J.; *et al*. Relation of circulating Matrix Gla-Protein and anticoagulation status in patients with aortic valve calcification. *Thromb. Haemost*. **2009**, *101*, 706–713.
9. Schurgers, L.J.; Barreto, D.V.; Barreto, F.C.; Liabeuf, S.; Renard, C.; Magdeleyns, E.J.; Vermeer, C.; Choukroun, G.; Massy, Z.A. The circulating inactive form of matrix gla protein is a surrogate marker for vascular calcification in chronic kidney disease: A preliminary report. *Clin. J. Am. Soc. Nephrol*. **2010**, *5*,
   568–575.
10. Rennenberg, R.J.; de Leeuw, P.W.; Kessels, A.G.; Schurgers, L.J.; Vermeer, C.; van Engelshoven, J.M.; Kemerink, G.J.; Kroon, A.A. Calcium scores and matrix Gla protein levels: Association with vitamin K status. *Eur. J. Clin. Invest*. **2010**, *40*, 344–349.
11. Ueland, T.; Dahl, C.P.; Gullestad, L.; Aakhus, S.; Broch, K.; Skardal, R.; Vermeer, C.; Aukrust, P.; Schurgers, L.J. Circulating levels of non-phosphorylated undercarboxylated matrix Gla protein are associated with disease severity in patients with chronic heart failure. *Clin. Sci.* **2011**, *121*, 119–127.
12. Schlieper, G.; Westenfeld, R.; Kruger, T.; Cranenburg, E.C.; Magdeleyns, E.J.; Brandenburg, V.M.; Djuric, Z.; Damjanovic, T.; Ketteler, M.; Vermeer, C.; *et al*. Circulating nonphosphorylated carboxylated matrix Gla protein predicts survival in ESRD. *J. Am. Soc. Nephrol*. **2011**, *22*, 387–395.
13. Shea, M.K.; O’Donnell, C.J.; Vermeer, C.; Magdeleyns, E.J.; Crosier, M.D.; Gundberg, C.M.; Ordovas, J.M.; Kritchevsky, S.B.; Booth, S.L. Circulating uncarboxylated matrix gla protein is associated with vitamin K nutritional status, but not coronary artery calcium, in older adults. *J. Nutr*. **2011**, *141*, 1529–1534.
14. Dalmeijer, G.W.; van der Schouw, Y.T.; Vermeer, C.; Magdeleyns, E.J.; Schurgers, L.J.; Beulens, J.W. Circulating matrix Gla protein is associated with coronary artery calcification and vitamin K status in healthy women. *J. Nutr. Biochem*. **2013**, *24*, 624–628.
15. Silaghi, C.N.; Fodor, D.; Craciun, A.M. Circulating matrix Gla protein: A potential tool to identify minor carotid stenosis with calcification in a risk population. *Clin. Chem Lab. Med*. **2013**, *51*, 1115–1123.
16. Dalmeijer, G.W.; van der Schouw, Y.T.; Magdeleyns, E.J.; Vermeer, C.; Verschuren, W.M.; Boer, J.M.; Beulens, J.W. Matrix Gla protein species and risk of cardiovascular events in type 2 diabetic patients. *Diabetes Care* **2013**, *36*, 3766–3771.
17. Dalmeijer, G.W.; van der Schouw, Y.T.; Magdeleyns, E.J.; Vermeer, C.; Verschuren, W.M.; Boer, J.M.; Beulens, J.W. Circulating desphospho-uncarboxylated matrix gamma-carboxyglutamate protein and the risk of coronary heart disease and stroke. *J. Thromb. Haemost*. **2014**, *12*, 1028–1034.
18. Van den Heuvel, E.G.; van Schoor, N.M.; Lips, P.; Magdeleyns, E.J.; Deeg, D.J.; Vermeer, C.; den, H.M. Circulating uncarboxylated matrix Gla protein, a marker of vitamin K status, as a risk factor of cardiovascular disease. *Maturitas* **2014**, 77, 137–141.
19. Mayer, O., Jr.; Seidlerova, J.; Bruthans, J.; Filipovsky, J.; Timoracka, K.; Vanek, J.; Cerna, L.; Wohlfahrt, P.; Cifkova, R.; Theuwissen, E.; *et al*. Desphospho-uncarboxylated matrix Gla-protein is associated with mortality risk in patients with chronic stable vascular disease. *Atherosclerosis* **2014**, *235*, 162–168.
20. Mayer, O., Jr.; Seidlerova, J.; Wohlfahrt, P.; Filipovsky, J.; Vanek, J.; Cifkova, R.; Windrichova, J.; Topolcan, O.; Knapen, M.H.; Drummen, N.E.; *et al*. Desphospho-uncarboxylated matrix Gla protein is associated with increased aortic stiffness in a general population. *J. Hum. Hypertens*. **2015**, doi:10.1038/jhh.2015.55.
21. Liu, Y.P.; Gu, Y.M.; Thijs, L.; Knapen, M.H.; Salvi, E.; Citterio, L.; Petit, T.; Carpini, S.D.; Zhang, Z.; Jacobs, L.; *et al*. Inactive matrix Gla protein is causally related to adverse health outcomes: A Mendelian randomization study in a Flemish population. *Hypertension* **2015**, *65*, 463–470.
22. Pivin, E.; Pruijm, M.; Ackermann, D.; Guessous, I.; Ehret, G.; Pechere-Bertschi, A.; Paccaud, F.; Mohaupt, M.; Vermeer, C.; Staessen, J.A.; *et al*. Inactive matrix Gla protein is associated with renal resistive index in a population-based study. *J. Hypertens*. **2015**, *33* (Suppl. S1), e15, doi:10.1097/01.hjh.0000467389.43721.16.
23. Pivin, E.; Ponte, B.; Pruijm, M.; Ackermann, D.; Guessous, I.; Ehret, G.; Liu, Y.P.; Drummen, N.E.; Knapen, M.H.; Pechere-Bertschi, A.; *et al*. Inactive matrix Gla-protein is associated with arterial stiffness in an adult population-based study. *Hypertension* **2015**, *66*, 85–92.
